# Infection point of care tests bridge the experience gap of antibiotic STOP decisions: clinicians versus students

**DOI:** 10.1093/jac/dkaf486

**Published:** 2026-03-07

**Authors:** Archit Singhal, Martine Nurek, Timothy Lau, James Mcentee, Luke Moore, Nabeela Mughal, Sonia Mason, Marcela Vizcaychipi, Suveer Singh

**Affiliations:** Faculty of Medicine, Imperial College London, London, UK; Faculty of Medicine, Imperial College London, London, UK; APMIC, Dept of Surgery and Cancer, Faculty of Medicine, Imperial College London, 369 Fulham road, London SW10 9NH, UK; Faculty of Medicine, Imperial College London, London, UK; Departments of Adult Intensive Care Medicine and Respiratory Medicine, Chelsea and Westminster Hospital NHS Foundation Trust, London, UK; Faculty of Medicine, Imperial College London, London, UK; Department of Microbiology and Infection, Chelsea and Westminster Hospital NHS Foundation Trust, London, UK; Faculty of Medicine, Imperial College London, London, UK; Department of Microbiology and Infection, Chelsea and Westminster Hospital NHS Foundation Trust, London, UK; Departments of Adult Intensive Care Medicine and Respiratory Medicine, Chelsea and Westminster Hospital NHS Foundation Trust, London, UK; Faculty of Medicine, Imperial College London, London, UK; APMIC, Dept of Surgery and Cancer, Faculty of Medicine, Imperial College London, 369 Fulham road, London SW10 9NH, UK; Faculty of Medicine, Imperial College London, London, UK; APMIC, Dept of Surgery and Cancer, Faculty of Medicine, Imperial College London, 369 Fulham road, London SW10 9NH, UK; Departments of Adult Intensive Care Medicine and Respiratory Medicine, Chelsea and Westminster Hospital NHS Foundation Trust, London, UK; Department of Adult Intensive Care Medicine, Royal Brompton Hospital, part of Guy’s and St Thomas’ Hospital NHS Foundation Trust, London, UK

## Abstract

**Background:**

Point of care tests (POCTs) offer accurate rapid diagnostics for infection, but not reduced antibiotic overuse in antibiotic stewardship (ABS) studies. Prescribing behaviour shaped by clinical experience may influence antibiotic decisions more than test performance. Understanding prescribing behavioural differences may inform ABS education.

**Objectives:**

To find out whether antibiotic decision making differ among medical students and intensive care clinicians when offered POCT use.

**Methods:**

An observational study depicted four simulated clinical vignettes of hospital acquired pneumonia. Clinicians and students decided to STOP or continue antibiotics, before and after a PCR-POCT result (negative for infection). Four clinico-biological (WBC/CRP) trajectories were tested: ‘clinical-biological improvement’, ‘clinical improvement/biological worsening’, ‘clinical worsening/biological improvement’ and ‘clinical-biological worsening’. STOP decisions, POCT requests and willingness to stop antibiotics were compared between groups using Chi-squared analysis, Wilcoxon-rank and logistic regression analyses.

**Results:**

Eighty-eight students and 70 clinicians participated. Pre-POCT, students stopped antibiotics less than clinicians (42% versus 53%, *P* = 0.007); most markedly in ‘clinical improvement/biological worsening’ (36% versus 73%, *P* < 0.001). Both groups requested POCT equally (65% versus 67%, *P* = 0.65). Negative POCT results raised student STOP rates to those of clinicians (70% versus 67%, *P* = 0.466); the greatest rise being in ‘clinical improvement/biological worsening’ (*P* = 0.006).

**Conclusions:**

Infection-detecting POCTs (negative) improved students’ antibiotic stop rates to the level of clinicians, particularly in cases of clinico-biological ambiguity. A requested and negative POCT result can reduce (over)cautious prescribing, especially with ambiguous trajectories. Such simulated clinical infection vignettes offer a learning tool to improve antimicrobial judgement, and confidence in POCT driven decision making.

## Background

Antimicrobial resistance (AMR) is a global health emergency.^1^ Approximately 700 000 people are estimated to be at clinical risk due to AMR per year, predicted to increase to 10 million by 2050.^2^ The impact may be 13-day average longer admissions, 8 million more annual hospital days and $20 billion loss globally.^3^

Healthcare factors known to drive AMR include overuse of antibiotics often with inappropriate prescribing.^3^ The burden of prescribing resides in the primary care, but treatment indication, agent choice and duration of therapy may be inappropriate up to 50% of the time in hospital.^4–7^ Within ICUs, 70% of patients are receiving antibiotics at any given time, despite no confirmed infection in up to half.^8,9^

ICUs are important environments for AMR due to high utility of invasive devices, broad-spectrum antibiotics, case severity dictating longer courses and shiftwork patterns.^10,11^ Furthermore, routine microbiology (RM) cultures fail to identify the pathogen in up to 50% of cases^12,13^ or are delayed due to processing by up to 48–72 h,^14–16^ often leading to unnecessary prolonged continuation of broad-spectrum antibiotics. Thus, there is a clinical need for quicker accurate identification of infections and better tools to determine when they have resolved. Furthermore, in recognition of the impact of AMR, organizational strategies, including the requirement for Microbiologist led ABS programmes in all acute UK NHS Trusts are an important health quality improvement initiative for some years.^17^

Rapid diagnostic infection point of care tests (herein abbreviated as POCT) are a developing part of ABS programmes (ASPs), their potential utility reaching wide public awareness during the COVID pandemic.^2,11^ These are rapid laboratory tests, identifying either an indirect biomarker of infection (e.g. IL1 or IL8) or a direct component of an infective agent [e.g. polymerase chain reaction (PCR) or metagenomics], providing results within hours.^18–21^ Therefore, POCTs can be useful in faster identification of infection, potentially enabling use of narrower-spectrum antibiotics from the start, earlier de-escalation during the treatment course, or cessation of antimicrobial treatment if no bacterial pathogens are found in suspected sepsis.^20,22^ In theory, usage of empirical antibiotic therapy could be reduced, although other aspects of prescribing behaviour will influence ASPs.

The utility of POCTs in antibiotic ‘start’ decisions has been well demonstrated, with PCR-based POCT for MRSA decreasing the time to appropriate antimicrobial therapy, hospital length of stay and treatment costs, as one example.^23^ However, its utility for ‘stop’ decisions is less clear cut, despite a high negative predictive value rendering it a highly efficacious rule-out test.^19,20^ The Ventilator-Associated-Pneumonia (VAP)-Rapid-2 study demonstrated that a rapid biomarker test (i.e. an indirect indicator of infection measuring bronchioloalveolar lavage fluid containing IL1 and IL8 in suspected VAP) was unable to reduce antibiotic-free days.^19^ This was not due to excellent test performance but rather clinician prescribing behaviour.^19^

Much of clinical decision making and behaviour is learned by experience (i.e. learned behaviour)^24^ and we expect this to hold true for POCT use and antibiotic stop decision making. We explored this as part of our previous study^25^ but results were inconclusive. Specifically, we found no evidence to suggest that years of experience exerts significant influence on POCT use or antibiotic stop decision making. However, the *post hoc* analysis was underpowered with unequal experience-related groups and inadequate distinction between degrees of experience.^25^

The potentially important role of experience in antibiotic stop decision making is scarcely investigated. We thus tested the antibiotic stop decisions of qualified clinicians, specialized in Intensive Care, Anaesthetics, Emergency Medicine, General Medicine and Infectious Disease (as collected in WHYSTOP study^25^) to those of medical students (data to be collected).

## Objectives

Given that students are naïve to post-qualification clinical experience and learned decision making, the study aims to shed light on (i) the role of learned behaviour in antibiotic prescribing decisions, (ii) how newer POCT tests influence judgements using conventional clinico-biological information and (iii) potential opportunities to modify behaviour with early (preclinical learning) interventions to improve antibiotic decision making.

### Hypotheses

Medical students lack experience and confidence in decision making in hospital settings, particularly in ICU.^26^ Thus, they may rely more on guidelines and tests such as POCTs for validation.^27^ We therefore hypothesized that:

- Students (versus clinicians) would be less inclined to stop antibiotics in cases of ICU respiratory infection.
- Students (versus clinicians) would be more inclined to seek additional investigations such as POCTs (to increase confidence in their decisions).
- Students (versus clinicians) would be more inclined to act in accordance with its results.

## Methods

### Participants

Medical students from the clinical training years 3–6 of Imperial College School of Medicine, Imperial College London, were contacted to take part through the College’s secure online portals using hyperlinks to the study website (hosted by XM Qualtrics, London, UK). Students of this institution have experienced sufficient teaching and ward-based clinical training, including intensive care medicine. We aimed to recruit the same number of participants (students) as our previous study (clinicians)^25^ i.e. (*N* = 70). A supplementary sample size calculation (using freeware G*Power v.3.1) confirmed that this would be sufficient to test our hypotheses (Appendix A, available as Supplementary data at JAC Online).

### Materials

We used the same four clinical vignettes as in Singh *et al.*^25^ (Appendix B), which depicted resolving/persisting or worsening VAP-infection after a course of antibiotics. The only amendment was the addition of normal ranges for biomarkers, deemed necessary during piloting (Appendix C). Each vignette comprised two types of data (clinical and biological, i.e. WBC, CRP), which were either improving or worsening. This returned four distinct trajectories (Table 1). The vignettes were representative cases of infection seen in critical care, and varying degrees of diagnostic uncertainty regarding the resolution of infection, commonly encountered.

**Table 1.** The four clinical vignettes used in this study

Vignette name	Description
Improvement	A post-operative case of a 66-year old man with bilateral pneumonia. Clinical and biological improvement after a 5-day course of antibiotics.
Worsening	A 65-year-old woman with lobar pneumonia who deteriorates, requiring mechanical ventilation. After initial stabilization and 4 days of antibiotics, there is a decline in clinical and biological status.
Discordant: clinically better, labs worse (‘disc clin better’)	A 62-year-old man with a severe lobar pneumonia requiring mechanical ventilation who improves clinically at 7 days and is extubated after an antibiotic course, but whose blood biomarkers have worsened.
Discordant: clinically worse, labs better (‘disc clin worse’)	A 54-year-old man with multilobar pneumonia who completes a course of antibiotics, is extubated but then deteriorates clinically despite improving blood biomarkers of infection.

Reproduced with permission from Singh *et al.*^25^.

Each vignette featured an infection related POCT, a PCR-based platform of a predefined bacterial organism panel.^20^ Participants were told that the POCT had high sensitivity and specificity, in line with laboratory-based diagnostic devices.^20^ The result of the POCT was always negative (suggesting no active lung infection), with one exception: in the improvement vignette only, participants were subsequently told that the negative POCT was erroneous (a laboratory error) and retesting gave a positive result, as previously.^25^

### Procedure

The procedure was identical to that used in the previous study.^25^ After providing informed consent, participants responded to all four vignettes presented in a random order except the improvement scenario, which was always presented last (to prevent the erroneous laboratory result from eroding POCT/laboratory credibility).

Per vignette, participants read introductory information (Table 1), made an antibiotic decision (stop/continue antibiotics), rated their confidence (1 = ‘not at all confident’, 6 = ‘extremely confident’) and indicated reason(s) for their choice (Appendix C). They were offered the POCT, which they could accept or reject, with a reason(s) for their choice (Appendix D). Irrespective of their choice (accept/reject POCT), a negative result was presented (those who declined the POCT were told that a colleague had performed it anyway). In light of this, participants were asked to update their treatment decision (stop/continue antibiotics) and confidence (1–6). In the improvement vignette, participants were then informed of the erroneous laboratory result and presented with a new, positive result; in response, they were asked to update their antibiotic decision and confidence. Finally, participants provided demographic information (Table 2).

**Table 2.** Demographic information of clinicians (*N* = 70) and students (*N* = 88)

	Clinicians [*n* (%)]	Students [*n* (%)]
Gender		
Male	36 (51%)	43 (49%)
Female	25 (36%)	25 (37%)
Prefer not to say	1 (1%)	0 (0%)
Missing	8 (11%)	20 (29%)
Total	70	88
Grade		
Consultant	18 (26%)	
SpR resident >3 years	16 (23%)	
SHO resident >1 year	17 (24%)	
FY resident <1 year	11 (16%)	
Missing	8 (11%)	
Year of medical school		
Year 3		13 (15%)
Year 4		35 (40%)
Year 5		15 (17%)
Year 6		5 (6%)
Missing		20 (23%)

SpR, specialist registrar; SHO, senior house officer; FY, foundation year.

### Analysis

Statistical analysis was conducted using IBM SPSS (version 28.0) and Stata/MP v.17, with graphs generated using Prism GraphPad (v.9.3.0). Cluster-adjusted chi-square analysis was used for dichotomous variables and outcomes. In this case, to compare the proportion of students with clinicians who: stopped antibiotics before receiving the POCT result (Hypothesis 1), requested POCT (Hypothesis 2) and stopped antibiotics after receiving the negative result (Hypothesis 3). Cluster-adjusted chi-square tests were used to compare pre- versus post-POCT decisions within each cohort.

Consistent with the previous study, we also computed a more sensitive measure of participants’ ‘willingness to stop’ antibiotics, by signing students’ ratings of confidence (1–6) in accord with their antibiotics decisions (positive if the decision was to stop antibiotics, negative if the decision was to continue).^25^ This returned a continuous measure of each student’s initial and final willingness to stop (WTS; −6 = minimal, 6 = maximal). We then explored the effect of patient trajectory [1 = improvement, 2 = ambiguous (discordant: clinically better, ‘disc clin better’ or discordant: clinically worse, ‘disc clin worse’), 3 = worsening], initial WTS (−6 = minimal, 6 = maximal) and desire for POCT (0 = POCT rejected, 1 = POCT accepted) on students’ final WTS (−6 = minimal, 6 = maximal), using a mixed-effects linear regression model (with a random intercept per participant and simultaneous entry of all predictors). The model was intentionally kept identical to the previous study, to facilitate a fair and meaningful comparison of results. No other predictors or covariates were tested.

### Approval

This study was approved by the Imperial College Research Ethics Committee (ICREC ref. 20IC6499).

## Results

### Sample characteristics

One hundred and two medical students accessed the survey. Of these, 24 (23%) did not complete a single vignette and were excluded from the study. Of the remaining 88, 13 completed one scenario (15%), five completed two (6%), one completed three (1%) and 69 completed all four (78%), yielding 302 scenario responses in total. In the clinician comparator group, of the 70 respondents 62 (89%) completed all four scenarios, two (3%) completed two scenarios and six (8%) completed one scenario; yielding 258 scenario responses in total. Demographic information for the two groups is shown in Table 2.

Hypothesis 1: Baseline (pre-POCT) prescribing behaviour.

**Figure 1.**
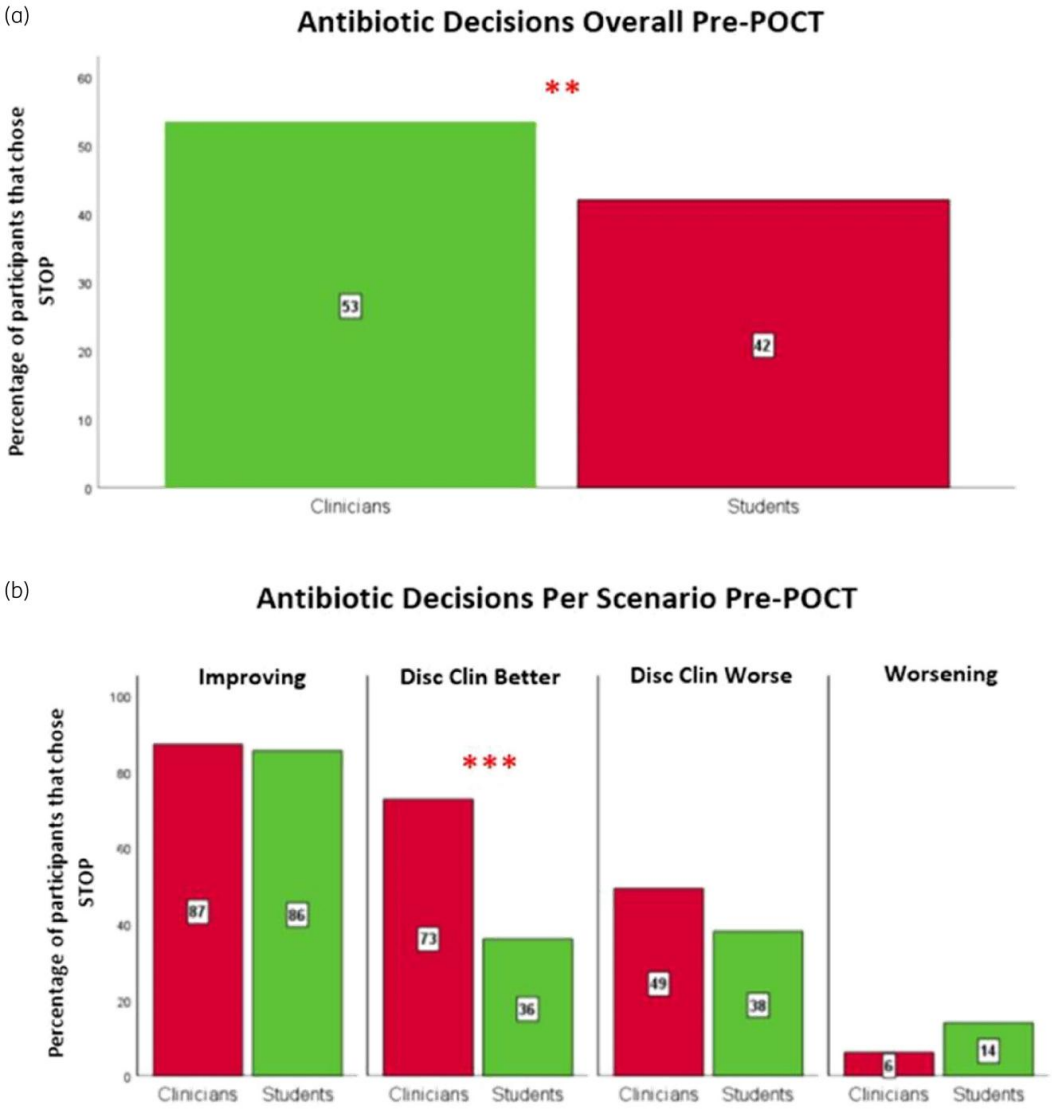
Comparison of the percentage of clinicians and students who chose to stop antibiotics before a negative POCT overall (a) and per scenario (b). The total number of clinician and student responses was *N*_Clin_ = 258 and *N*_Stu_ = 302; *n*_Clin_ = 62 and *n*_Stu_ = 69 in improving, *n*_Clin_ = 66 and *n*_Stu_ = 75 in disc clin better, *n*_Clin_ = 65 and *n*_Stu_ = 79 in disc clin worse, and *n*_Clin_ = 65 and *n*_Stu_ = 79 in worsening. Red bars represent clinicians and green bars represent students. Differences in pre-POCT stopping rates between the two groups (clinicians versus students) were analysed using cluster-adjusted chi-square analysis. Significance denoted at: *P < 0.05, **P < 0.01, ***P < 0.001.

Overall, students were significantly less likely than clinicians to stop antibiotics at baseline [i.e. before receiving the negative POCT: 42% (127/302) versus 53% (138/258); *P* = 0.007, Figure 1a]. In detail per scenario (Figure 1b), the improvement and worsening scenarios had high and low stopping rates, respectively, for students and clinicians equally (improvement, *P* = 0.792; worsening, *P* = 0.129). Students were less likely to stop in the discordant scenarios but only significantly less so in the disc clin better scenario [disc clin better 36% (27/75) versus 73% (48/66), *P* < 0.001; not in the disc clin worse scenario 38% (30/79) versus 49% (32/65), *P* = 0.180]. Indeed, clinicians and students disagreed in this scenario, with most students electing to continue and most clinicians electing to stop. Similar results were obtained for the WTS variable (Appendices E and F).

Hypothesis 2: The frequency of POCT requests between students and clinicians.

**Figure 2.**
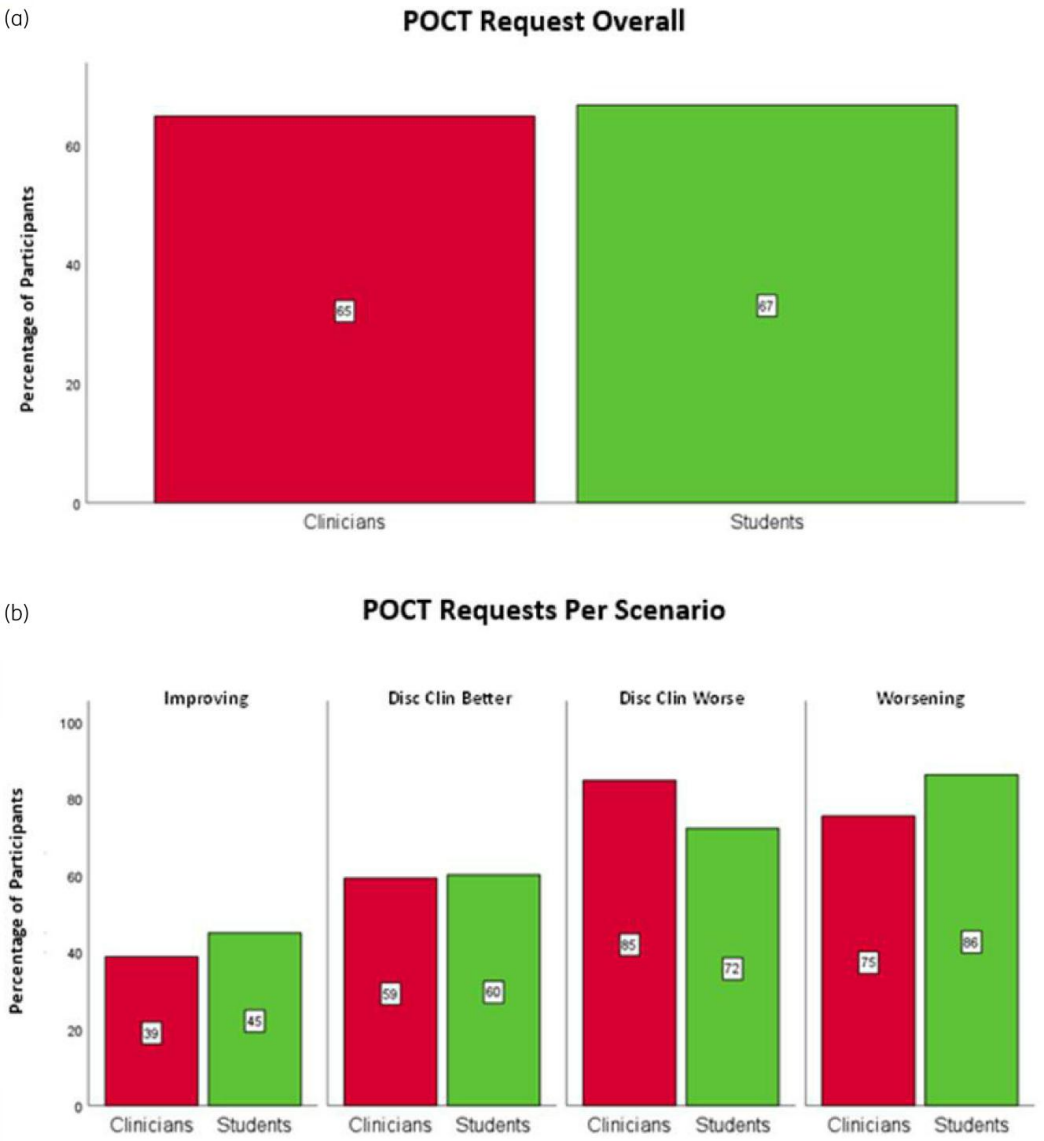
Percentage of clinicians and students who requested a POCT overall (a) and per scenario (b). The total number of clinician and student responses was *N*_Clin_ = 258 and *N*_Stu_ = 302; *n*_Clin_ = 62 and *n*_Stu_ = 69 in improving, *n*_Clin_ = 66 and *n*_Stu_ = 75 in disc clin better, *n*_0_ = 65 and *n*_Stu_ = 79 in disc clin worse and *n*_Clin_ = 65 and *n*_Stu_ = 79 in worsening. Red bars represent clinicians and green students. Differences in proportions (clinicians versus students) were analysed using chi-square analysis (clustered adjusted when computed overall). Significance denoted at: *P < 0.05, **P < 0.01, ***P < 0.001.

Overall, and contrary to Hypothesis 2, a similar proportion of students [65% (167/258)] versus clinicians [67% (201/302)] requested the POCT, overall (*P* = 0.650, Figure 2a) and per scenario (all *P* ≥ 0.073, Figure 2b).

Hypothesis 3: The frequency of final (post-POCT) prescribing stop decisions.

**Figure 3.**
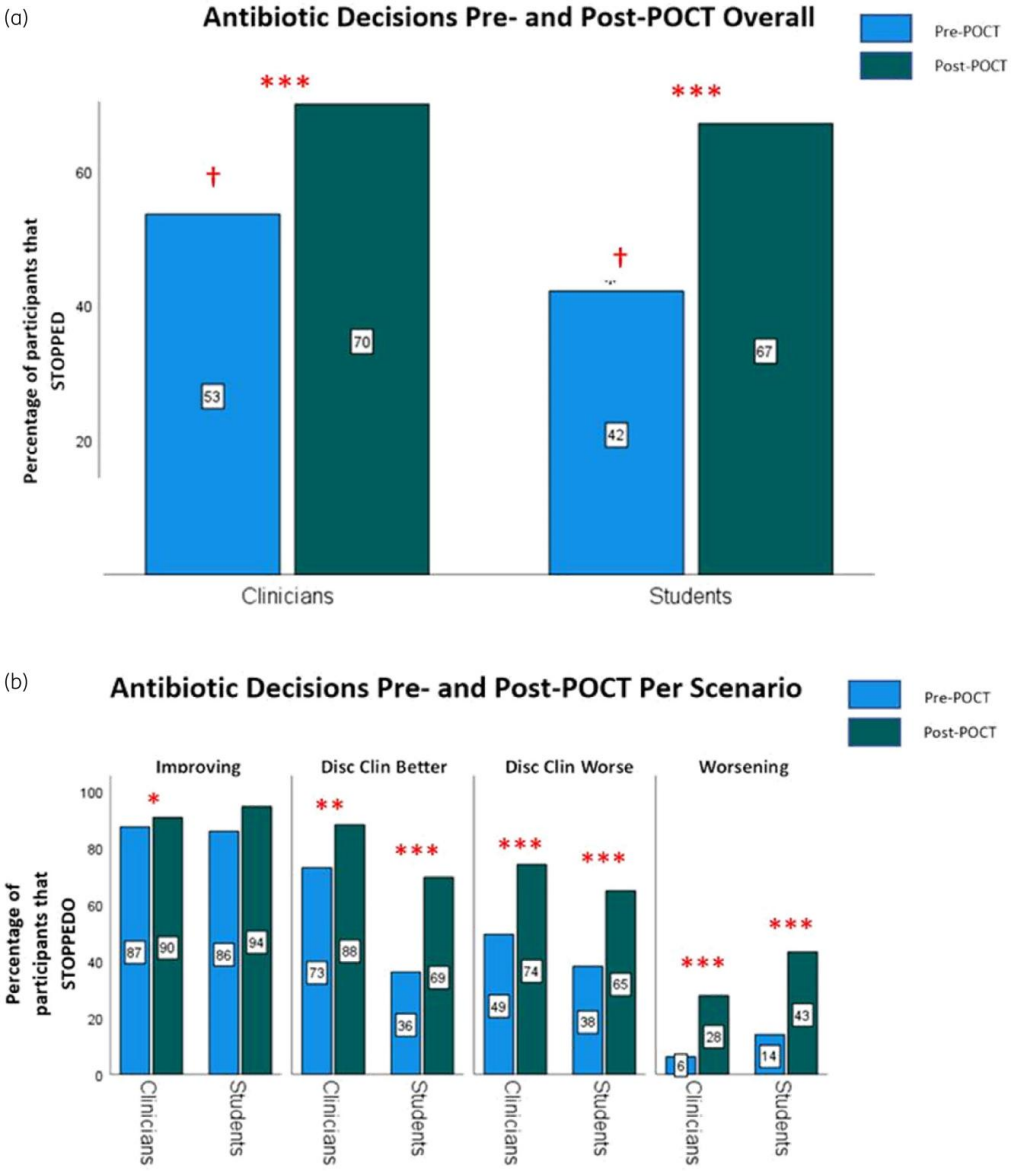
Pre-POCT versus post-POCT stop decisions overall (a) and per scenario (b) among clinicians and students. The total number of clinician and student responses was *N*_Clin_ = 258 and *N*_Stu_ = 302; *n*_Clin_ = 62 and *n*_Stu_ = 69 in improving, *n*_Clin_ = 66 and *n*_Stu_ = 75 in disc clin better, *n*_Clin_ = 65 and *n*_Stu_ = 79 in disc clin worse and *n*_Clin_ = 65 and *n*_Stu_ = 79 in worsening. Blue bars/boxes indicate pre-POCT responses, green bars show post-POCT responses. Significance denoted at: *P < 0.05, **P < 0.01, ***P < 0.001. **^†^**Denotes difference in stopping rate between clinicians versus students pre-POCT at *P* < 0.01. (a) Percentage of clinicians and students who chose to stop antibiotics before versus after a negative POCT result. Differences in proportions were analysed using cluster-adjusted chi-square tests. (b) Percentage of clinicians and students who chose to stop antibiotics before versus after a negative POCT result, per scenario. Intragroup differences (pre-POCT versus post-POCT stopping) and intergroup.

Overall, and in contrast to Hypothesis 3, clinicians and students were equally likely to stop antibiotics following receipt of a negative POCT result [students versus clinicians; 67% (202/302) versus 70% (180/258); *P* = 0.466]. As Figure 3a shows, a negative POCT result significantly increased the frequency of stop decisions for both clinicians [53% (138/258) pre-POCT versus 70% (180/258) post-POCT; *P* < 0.001] and students [42% (127/302) pre-POCT versus 67% (202/302) post-POCT; *P* < 0.001], although the rate of change was higher for students due to their lower baseline (pre-POCT) stop rate.

These intragroup increases in stop decisions post-POCT held true per scenario (Figure 3B), except for clinicians in the improvement scenario [where the stop rate was high at baseline: 90% (56/62) pre-POCT versus 94% (65/69) post-POCT; *P* = 0.404]. In the disc clin better scenario, a negative POCT increased stop decisions more for students [36% (27/75) to 69% (52/75); *P* < 0.001] than clinicians [73% (48/66) to 88% (58/66); *P* = 0.006] (Figure 3b). In the disc clin worse scenario (Figure 3b), a negative POCT increased the inclination to stop antibiotics similarly in both groups changing the majority decision from continuation (pre-POCT) to stop [clinicians; 49% (32/65) versus 74% (48/65), *P* < 0.001; students 38% (30/79) versus 65% (51/79), *P* < 0.001]. In the worsening scenario, where initial decisions strongly favoured continuation in both groups (students STOP = 14% (11/79), clinicians STOP = 6% (4/65)], a negative POCT result increased the stop rate to similar extents (students STOP = 43% (34/79), clinicians STOP = 28% (18/65)], but did not shift the majority decision from continuation to cessation in either group. WTS data were comparable, and are reported in Appendices E and F.

### Effect of patient trajectory, initial WTS and in/voluntary receipt of POCT result on final WTS

Mixed-effects linear regression suggested that final (post-POCT) WTS was a function of the patient’s trajectory: students were significantly less inclined to stop antibiotics (despite receiving a negative POCT) when the patient’s trajectory was ambiguous (discordant scenarios) or worsening, as opposed to improving [*b* = −0.74 (−1.26, −0.23), *P* = 0.005]. This is consistent with our previous study of clinicians.^25^ Final WTS was also a function of the student’s initial leaning, with high (low) WTS pre-POCT predicting high (low) WTS post-POCT [*b* = 0.51 (0.42, 0.61), *P* < 0.001], again concurrent with clinicians.^25^ Finally, the manner in which the POCT result was acquired (voluntarily versus involuntarily) did not influence students’ final WTS [*b* = 0.60 (−0.10, 1.30), *P* = 0.095]. This lies in contrast to clinicians who were significantly more willing-to-stop when POCT was actively requested versus passively received.^25^ These results did not change when final WTS was treated as ordinal rather than linear (Appendix G).

### Effect of a positive POCT result on the confidence in stop prescribing decision

After learning that the POCT result had changed from negative to positive (improvement only scenario), the stop rate decreased significantly for clinicians [90% (56/62) versus 61% (38/62), *P* < 0.001] and students [94% (65/69) versus 45% (31/69)] as did WTS (Appendix H).

### Reasons for antibiotic decisions between students and clinicians

**Figure 4.**
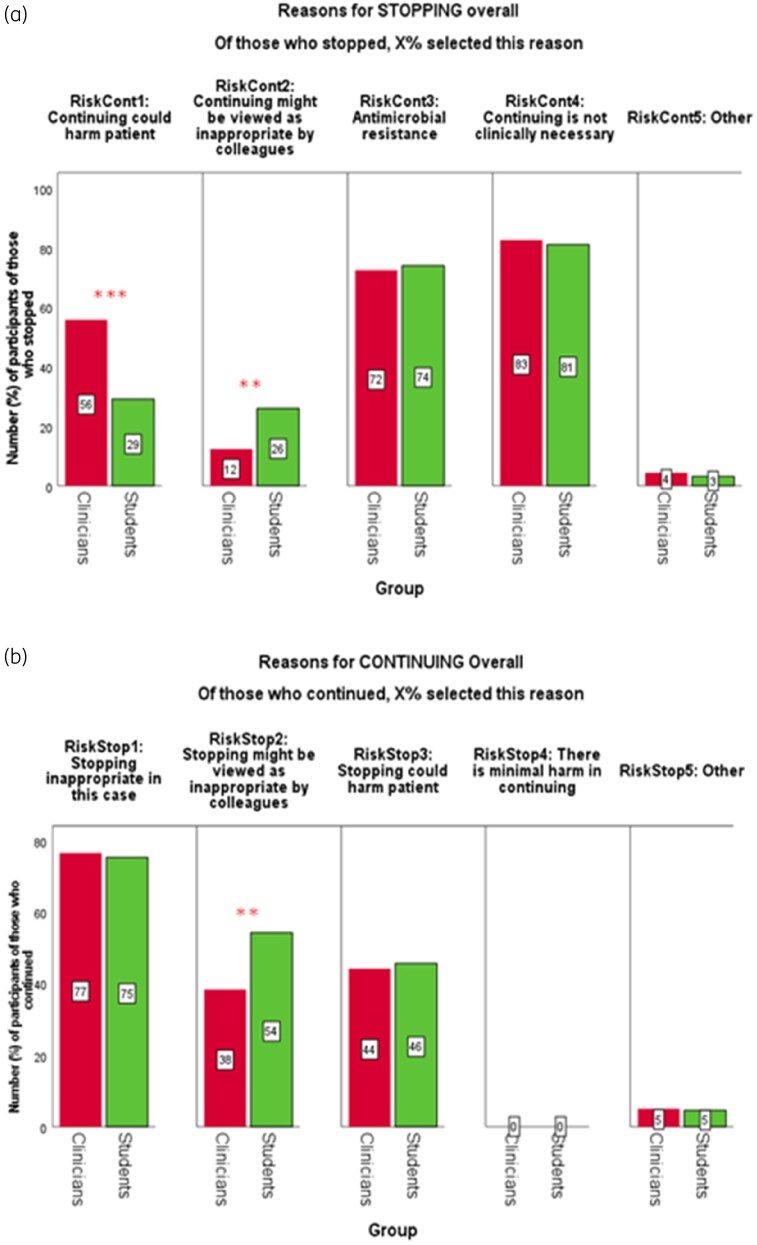
Percentage of clinicians (*N*_Clin_ = 258) and students (*N*_Stu_ = 302) who selected each reason for stopping (a) or continuing (b) antibiotics, overall. Red bars represent clinicians and green bars represent students. Participants could select multiple reasons for their decision. Differences in proportions were analysed using cluster-adjusted chi-square analysis. Significance denoted at: *P < 0.05, **P < 0.01, ***P < 0.001.

Students were significantly less likely to consider patient harm occurring by not stopping antibiotics than were clinicians (Figure 4), whereas students were significantly more likely to consider negative feedback by peer review as a reason that influenced their antibiotic decision than were clinicians (Figure 4).

### Reasons for rejecting and requesting POCT

**Figure 5.**
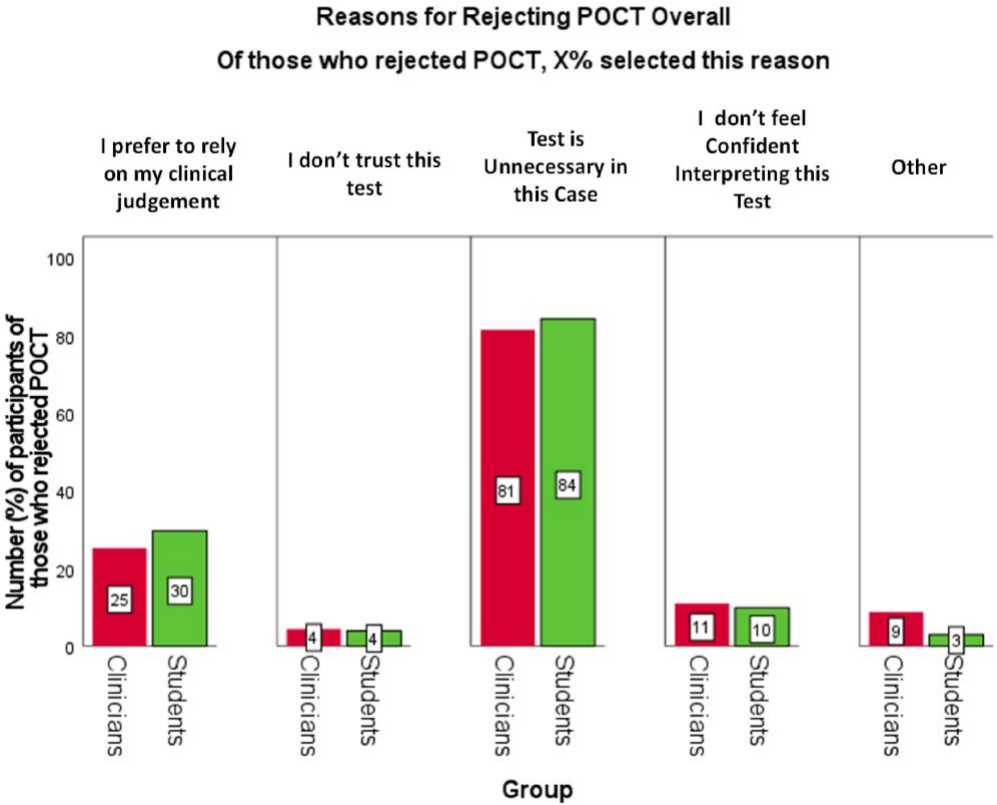
Percentage of clinicians (*N*_Clin_ = 258) and students (*N*_Stu_ = 302) who selected each reason for rejecting POCT. Red bars represent clinicians and green bars represent students. Participants could select multiple reasons for their decision. Differences in proportions (clinicians versus students) were analysed using cluster-adjusted chi-square analysis. There were no significant differences.

The most frequent reason for rejecting POCT (>80%) was the test was deemed unnecessary to influence the antibiotic decision. The next frequent answer (∼20–25%) was the preference to use their own clinical judgement. There was no difference between students or clinicians in their reasons for rejecting POCT (Figure 5).

**Figure 6.**
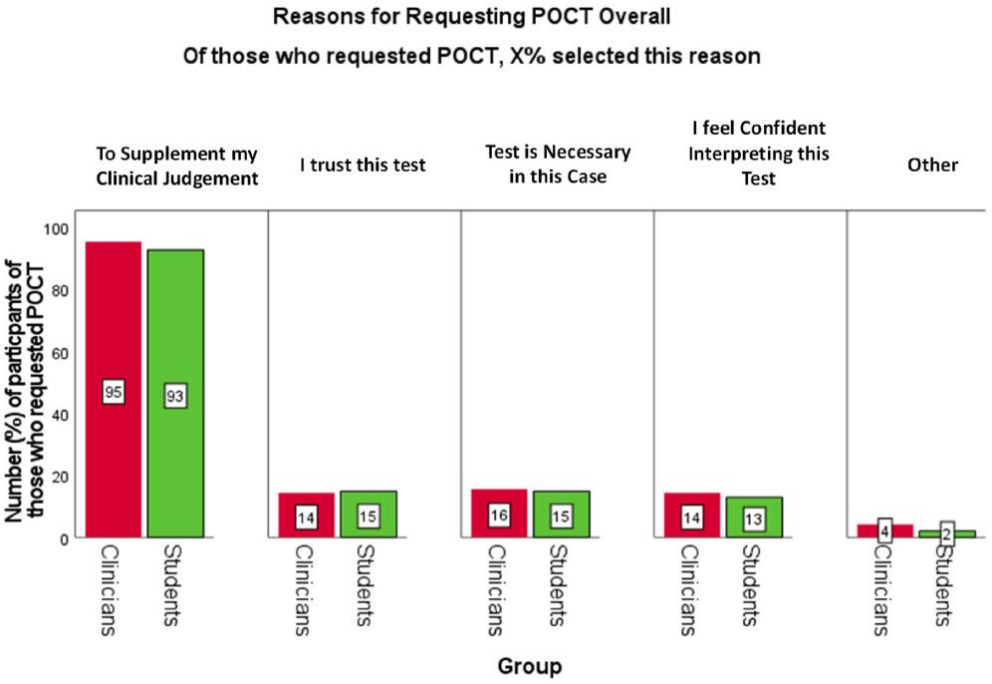
Percentage of clinicians (*N*_Clin_ = 258) and students (*N*_Stu_ = 302) who selected each reason for requesting POCT. Red bars represent clinicians and green bars represent students. Participants could select multiple reasons for their decision. Differences in proportions (clinicians versus students) were analysed using cluster-adjusted chi-square analysis. There were no significant differences.

The overwhelming majority expressed reason to request POCT was to support clinical judgement in making antibiotic decision (>90% of participants who accepted it). Once again, there was congruence between students and clinicians (Figure 6).

Details of the reasons for stopping/continuing antibiotics, and rejecting or accepting the offer of a POCT test, per scenario are displayed in Appendices I–L.

## Discussion

We investigated the effect of clinical experience on how POCT use could influence antibiotic prescribing decisions for ICU-related infection. Further, how prescribing behaviour changed with degrees of clinical uncertainty (after resolution of infection following a completion course of antibiotics) was addressed by varying the clinico-biological trajectories of the case-based vignettes presented. Participants made an antibiotic decision (stop/continue) before and after receiving a POCT result (negative), irrespective of whether they requested a POCT. Students were significantly less likely than clinicians to stop antibiotics at baseline (i.e. before the POCT result), particularly in ambiguous scenarios featuring discordant information. Professional development moves through a spectrum from ‘novice’ to ‘expert’ by forming a ‘mental library’ of past experiences—direct or anecdotal—that inform their decision making.^24,27,28^ Without this ‘library’, novice decision making is usually cautious and risk averse.^24,27,29^ Thus, in the face of uncertainty, it is unsurprising that students adopt protective prescribing, as defined by the necessity concerns framework, due to the perceived risk of undertreating the patient.^30^ In scenarios of discordant clinical and biological trajectory, students were less inclined than clinicians to STOP, even with clinical improvement (but biological worsening). Thus, clinicians appear to prioritize clinical (over biological) data, while students appear to give them equal weight. Clinicians it would seem, ‘trust’ clinical trajectory more than biological, when faced with uncertainty regarding their antibiotic prescribing judgements. This reflects the historical evolution of clinical medicine, with laboratory-based findings incorporated into clinical practice later during William Osler’s era.^31^ On the contrary, students maintained equipoise for the influence of clinical and biological trajectories, more often opting to continue given a worsening trend of either. Clinicians’ preference for clinical data may be ‘learned behaviour’^32^ and/or a heuristic (i.e. ‘pattern recognition’).^33^ Over years of experience, they learn to associate clinical deterioration with ongoing infection, superseding inferred biological biomarker results.

We expected students’ inexperience to trigger more POCT requests (relative to clinicians), as an additional arbiter to their judgement during uncertainty, and so reduce the chance of judgement ‘error’.^27^ Indeed, novices/students are generally more reliant on test results, to seek validation/justification for their decisions.^24,27^ Clinicians, on the other hand, might be distrusting of POCT,^34,35^ for various reasons, leading to fewer POCT requests. Contrary to this hypothesis, two-thirds of students and clinicians requested the POCT with equal frequency in all scenarios, and more so in scenarios of clinical deterioration (disc clin worse and worsening). Notably, participants were reassured a priori of the validity and reliability of the test, which may have increased trust in POCT among clinicians. This in and of itself might indicate justification for, and practical importance of providing infection POCT platforms when trying to decide whether an infection has resolved or not.

A negative POCT predictably increased STOP rates: more so among students (less inclined to stop pre-POCT) than clinicians (more inclined to stop pre-POCT), leading to similar frequency of post-POCT stop decisions across groups. POCT may thus have helped students to overcome their initially cautious approach (‘protective prescribing’), nudging their decision making closer to that of an experienced clinician. It is possible, therefore, that a negative POCT nudge may have matched learned behaviour for this specific decision-making task.

Interestingly, how the POCT result was acquired—voluntarily versus involuntarily—influenced clinicians’ but not students’ antibiotic decisions. Although equally requested, clinicians were more likely to change to a STOP decision if it was actively requested (versus passively received) whereas students did not appear to differentiate between the two. Thus, clinicians seemed to request a POCT tactically, on its expected utility/perceived appropriateness, whereas students appeared accepting of the POCT and its result at face value of utility. Students tend to abide by protocols,^24^  ^,27^ more so than clinicians who were more likely to deviate from them. Furthermore, they may be more attuned to feedback, correction and new information.^27,36^

### Implications

Presenting students with a negative POCT result appears to reduce (over)cautious antibiotic prescribing, approaching that of experienced clinicians. Simulated vignettes of clinical infection incorporating POCT diagnostics could thus offer a promising learning tool to develop students’ prescribing decision-making behaviour and expedite experiential learning. Vignettes depicting ambiguity (here operationalized as discordance between clinical and biological data) may be particularly useful to students, who lack confidence dealing with uncertainties.^37^ It can be argued some medical school curricula do not adequately provide for such training in decision making during clinical uncertainty.^37^ As such real patient modelling with embedded ambiguity and uncertainty can provide safe and low-stakes simulation training, while educating about the utility of emerging rapid diagnostic technologies such as infection POCTs to inform and improve antibiotic judgements. Indeed, case-based learning has been shown to deepen learning by encouraging analytical (‘Type 2’) reasoning.^38^ To function effectively as a learning tool, vignettes should include immediate feedback (based on real patient responses or outcomes); this would allow students to learn, through trial and error, the situations in which POCTs are most gainfully used. Effective use of the concept and potential value of infection POCTs in the early stages of medical education could also promote ‘good habits’, producing a cohort of clinicians who are confident incorporating novel diagnostics into antibiotic decision making. The long-term effects of such early educational interventions, offered serially with regular formative review, to improve antimicrobial stewardship and prescribing culture warrant high priority in future research.

### Strengths and limitations

To our knowledge, this the first study to measure (and therefore quantify) experience-related differences in antibiotic stop decisions in this context. While previous studies have identified cognitive-behavioural factors influencing antibiotic decisions none have quantitively measured the effect of experience.^25,30,39,40^

However, the generalisability of these findings to real-life, high-stakes ICU settings has not been tested. It may be too easy to stop antibiotics in a hypothetical scenario, with no real-time consequences for prescriber or patient. Similarly, it may be too easy to request a POCT without considerations of resource and financial strains.^41^

It is also important to note that our vignettes were simplistic, omitting other investigations usually conducted, such as imaging or other biomarkers for infection, e.g. procalcitonin.^42,43^ This was intentional, as the predictive accuracy of simple models is usually reduced by adding complexity.^44^ Relatedly, we did not offer participants the option to change or de-escalate antibiotics, lest it became the ‘safest’, thus default option, particularly in the ambiguous scenarios. Therefore, some of the increases in stop decisions may not necessarily reflect a desire to stop antibiotics completely, but rather they would not continue with the same course. These factors limit the generalisability of the results. Present findings would benefit from replication in more complex vignettes that provide the option to de/escalate and in real-time clinical settings, both of which are currently underway. ^45,46^

## Take home messages

- Experience influences how students and clinicians decide when to stop antibiotics.
- Students often lack confidence in antibiotic prescribing, especially with uncertain clinical trajectories.
- POCT may help build confidence and guide decision making.
- Case-based learning with uncertain scenarios, POCT options and timely feedback could prepare students for real clinical ambiguity.
- Such training also familiarizes students with emerging diagnostic POCT tools.
- The effectiveness and long-term impact of this simulated learning approach on prescribing culture still need evaluation.

## Supplementary Material

dkaf486_Supplementary_Data

## Data Availability

The datasets used and/or analysed during the current study are available from the corresponding author on reasonable request.

## Supplementary data

Appendices A to L are available as Supplementary data at JAC Online.
