# Supplementary material for: Infection point of care tests bridge the experience gap of antibiotic STOP decisions: clinicians versus students
Source: J Antimicrob Chemother. 2026 Mar 7;81(4):dkaf486. doi: 10.1093/jac/dkaf486 (PMC13016884; doi:10.1093/jac/dkaf486)

**Appendix**

**A. Power Calculation**

To ensure that this would provide sufficient power to test our hypotheses, we also performed a sample size calculation using G*Power. This revealed that 349 responses were needed to detect a small-to-medium effect (phi/Cohen’s w=0.15) in a chi-square analysis comparing the frequency of stop decisions among students vs. clinicians  (power=80%, alpha=0.05,  df=1). As each participant gave 4 responses, we also computed the “design effect”(35) using the formula 1+(n–1)ρ, where n is the cluster size (4) and ρ the intraclass correlation (or Cronbach’s alpha36) from the previous study23 (0.061). Multiplying the number of required responses (349) by the design effect (1.183) suggested that 413 responses were needed (349 x 1.183). At 4 responses per participant, 103 participants were required in total (52 students and 52 clinicians). Our projected sample size of 70 students (present study) and 70 clinicians (previous study) would therefore be sufficient.

**B. The 4 clinical vignettes**

*Normal ranges only shown to medical students.

**The “improvement” vignette**

*Details of admission:*

*A 66-year-old male with a background of diverticulitis was admitted for elective sigmoid colectomy. Four days post-operatively he developed worsening hypoxia and pyrexia. His observations were the following:*

| *Respiratory rate* | *24* (12-20) |
| --- | --- |
| *SpO2* | *91% on room air* (>95%) |
| *Heart rate* | *90* (51-90) |
| *Blood pressure* | *102/71 mmHg* (120/80) |
| *Temperature* | *38.1 C*  (36.1-38.0) |

*There was bilateral pulmonary consolidation on both chest radiograph and a CT pulmonary angiogram, CTPA and nil else. His blood tests demonstrated a WBC of 16 (4.0-11.0) and CRP of 98 (0-10). He was empirically started on co-amoxiclav.*

*Five days later:*

*5 days later, he had improved shortness of breath and was afebrile. His observations were:*

| *Respiratory rate* | *18* (12-20) |
| --- | --- |
| *SpO2* | *98% on room air* (>95%) |
| *Heart rate* | *83* (51-90) |
| *Blood pressure* | *112/70 mmHg* (120/80) |
| *Temperature* | *37.2 C*  (36.1-38.0) |

*He was eating and drinking well and mobilising on the ward. His repeat blood tests demonstrated a WBC of 10 (4.0-11.0) and CRP of 25 (0-10).*

**The “worsening” vignette**

*Details of admission:*

*A 65-year-old female was admitted with a 2-day history of worsening shortness of breath and a non-productive cough. She has no past medical history. She was penicillin allergic. Her admission observations were:*

| *Respiratory rate* | *22* (12-20) |
| --- | --- |
| *SpO2* | *84% on room air* (>95%) |
| *Heart rate* | *98* (51-90) |
| *Blood pressure* | *104/65 mmHg* (120/80) |
| *Temperature* | *37.9 C*  (36.1-38.0) |

*There was right basal consolidation on her chest radiograph. Her blood tests demonstrated a WBC of 14 (4.0-11.0) and a CRP of 102 (0-10). She was empirically started on levofloxacin and clarithromycin. Within 24 hours she deteriorated and required mechanical ventilation.*

*Three days later:*

*3 days later, after an initial improvement in ventilation, she became febrile. Her observations were:*

| *Respiratory rate* | *25* (12-20) |
| --- | --- |
| *SpO2* | *92% on FiO2 21%* (>95%) |
| *Heart rate* | *120* (51-90) |
| *Blood pressure* | *130/70 mmHg* (120/80) |
| *Temperature* | *39.0 C*  (36.1-38.0) |

*Her repeat blood tests demonstrated WBC 18 (4.0-11.0) and CRP 132 (0-10).*

**The discordant vignette with clinical improvement but biochemical worsening (“disc clin better”):**

*Details of admission:*

*A 62-year-old male was admitted to ITU with a 4-day history of pyrexia, shortness of breath, and a productive cough with rusty sputum. He has a past medical history of type 2 diabetes and hypertension. His observations were the following:*

| *Respiratory rate* | *28* (12-20) |
| --- | --- |
| *SpO2* | *86% on room air* (>95%) |
| *Heart rate* | *125* (51-90) |
| *Blood pressure* | *104/64 mmHg* (120/80) |
| *Temperature* | *38.1 C*  (36.1-38.0) |

*A chest radiograph demonstrated left basal consolidation. His blood tests demonstrated a WBC of 14 (4.0-11.0) and a CRP of 70 (0-10). Sputum culture grew fully sensitive Klebsiella Pneumonia. He was intubated and ventilated and empirically started on piperacillin/tazobactam. His initial blood gas findings were:*

*·*  *FiO2 0.6*

*·*  *PaO2 of 7.9 kPa*  (10.7-13.3)

*·*  *PaCO2 5.5 kPa*  (4.7-6.0)

*· Base excess of -5* (-2 to +2)

*Seven days later:*

*7 days into his admission, he was improving on ventilation and he is extubated and weaned onto room air. He was feeling much better. His observations were:*

| *Respiratory rate* | *16* (12-20) |
| --- | --- |
| *SpO2* | *94% on room air* (>95%) |
| *Heart rate* | *85* (51-90) |
| *Blood pressure* | *110/70 mmHg* (120/80) |
| *Temperature* | *37.4 C*  (36.1-38.0) |

*His repeat blood tests demonstrated a WBC of 16 (4.0-11.0) and a CRP of 110 (0-10).*

**The discordant vignette with clinical worsening but biochemical improvement (“disc clin worse”):**

*Details of admission:*

*A 54-year-old male was admitted to ITU with a 2-day history of pyrexia, shortness of breath and a productive cough. He has no past medical history. His observations were the following:*

| *Respiratory rate* | *22* (12-20) |
| --- | --- |
| *SpO2* | *88% on room air* (>95%) |
| *Heart rate* | *130* (51-90) |
| *Blood pressure* | *115/54 mmHg* (120/80) |
| *Temperature* | *38.1 C*  (36.1-38.0) |

*A chest radiograph demonstrated bilateral pulmonary infiltrates. His blood tests demonstrated a WBC of 19 (4.0-11.0) and a CRP of 110 (0-10). Sputum culture grew fully sensitive to streptococcus pneumonia. He was intubated and ventilated and empirically started on piperacillin/tazobactam. His initial blood gas findings were:*

· FiO2 0.6

· PaO2 of 7.9 kPa (10.7-13.3)

· PaCO2 4.5 kPa (4.7-6.0)

· Base excess of -5 (-2 to +2)

*Five days later:*

*5 days into his admission, he was extubated and weaned onto room air. Chest radiograph findings were unchanged. A few days following extubation, he developed new pyrexia and increased oxygen requirement.*

*Seven days later:*

*7 days into his admission, his observations were:*

| *Respiratory rate* | *20* (12-20) |
| --- | --- |
| *SpO2* | *92% on FiO2 0.6* (>95%) |
| *Heart rate* | *100* (51-90) |
| *Blood pressure* | *130/70 mmHg* (120/80) |
| *Temperature* | *37.8 C*  (36.1-38.0) |

*His repeat blood tests demonstrated a WBC of 9 (4.0-11.0) and a CRP of 24 (0-10).*

The *improvement* and *worsening* vignettes served as controls, in that they clearly supported a decision to stop and continue antibiotics respectively. The discordant scenarios, *disc clin better* and *disc clin worse,* were designed to present a greater diagnostic challenge with likely equipoise in the inclination to stop/continue due to either clinical or biological factors deteriorating. Hence, these two scenarios allowed us to explore the hierarchy of importance (if any) between clinical and lab-based biological trajectory in driving stop decisions amongst different cohorts.

**C. Reasons for Stopping and Continuing Antibiotics**

| **Reasons for STOPPING antibiotics** | **Reasons for CONTINUING antibiotics** |
| --- | --- |
| *Clinicians that elected to STOP antibiotics were presented with the following list of reasons and asked to tick all that apply:* | *Clinicians that elected to CONTINUE antibiotics were presented with the following list of reasons and asked to tick all that apply:* |
| Continuing antibiotics could harm the patient;  Continuing antibiotics might be viewed as inappropriate by colleagues;  Continuing antibiotics is not clinically necessary based on the information provided;  Continuing antibiotics poses population risks (i.e., antimicrobial resistance);  Other (if selected, the participant was asked to elaborate using free text). | Stopping antibiotics could harm the patient;  Stopping antibiotics might be viewed as inappropriate by colleagues;  Stopping antibiotics is inappropriate based on the clinical information provided;  There is relatively minimal harm in continuing antibiotics;  Other (if selected, the participant was asked to elaborate using free text). |

***Appendix C. List of reasons presented to clinicians that elected to stop antibiotics (left) or continue antibiotics (right).*** *C*linicians could select as many as needed. Reproduced with permission from Singh et al. (under review).

**D. Reasons for Performing or Not Performing the POCT**

| **Reasons for PERFORMING POCT** | **Reasons for NOT PERFORMING POCT** |
| --- | --- |
| *Clinicians that chose to perform the POCT were presented with the following list of reasons and asked to tick all that apply:* | *Clinicians that chose NOT to perform the POCT were presented with the following list of reasons and asked to tick all that apply:* |
| To supplement my clinical judgment;  I trust this test;  The test is necessary in this case;  I feel confident interpreting this test;  Other (if selected, the participant was asked to elaborate using free text). | I prefer to rely on my clinical judgement;  I do not trust the test;  This test is unnecessary in this case;  I don’t feel confident interpreting this test;  Other (if selected, the participant was asked to elaborate using free text). |

***Appendix D. List of reasons presented to clinicians that accepted (left) vs. rejected (right) the POCT****.* Clinicians could select as many as needed. Reproduced with permission from Singh et al. (under review).

**E. Pre and Post POCT WTS Overall**


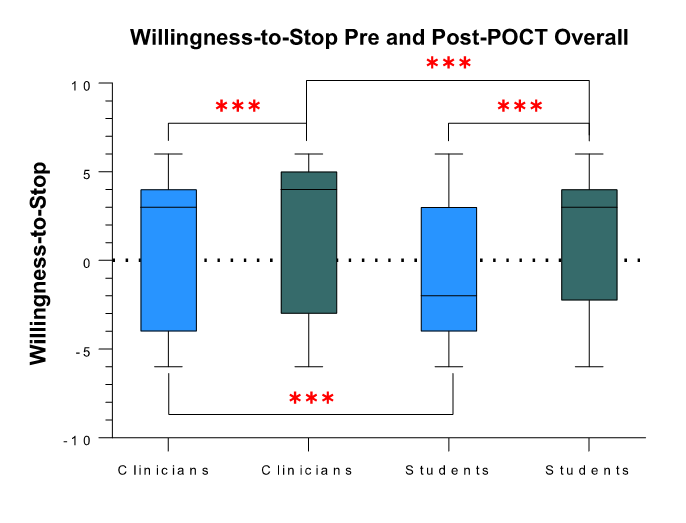


***Appendix E. Pre-POCT vs. post-POCT WTS amongst clinicians (N_Clin_=258) and students (N_Stu_=302), overall.*** Blue bars/boxes indicate pre-POCT responses, green post-POCT responses. Pre-POCT vs. Post-POCT Significance denoted at: *p<0.05, **p<0.01, ***p<0.001.

WTS1 and WTS2 were not normally distributed (Shapiro-Wilk statistic (W)=0.840, df=560, p<0.001), therefore intragroup differences (pre-POCT vs. post-POCT) were analysed using Wilcoxon-Signed Rank tests and inter-group differences (clinicians vs. students) were analysed using Mann-Whitney U-tests.

**F. Pre and Post POCT Per Scenario**


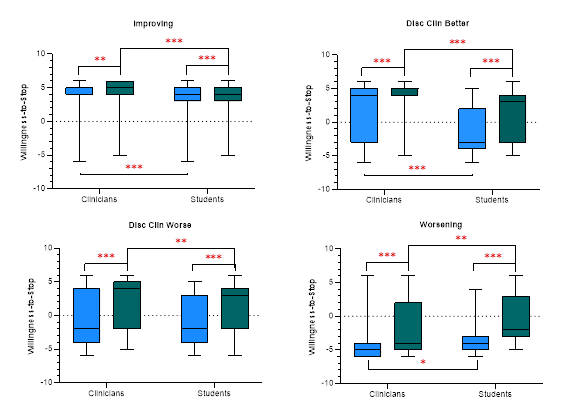


***Appendix F. Pre-POCT vs. post-POCT WTS amongst clinicians and students, per scenario.*** Per scenario responses: *n*_Clin_=62 and *n*_Stu_= 69 in *improving*, *n*_Clin_=66 and *n*_Stu_= 75 in *disc clin better*, *n*_Clin_=65 and *n*_Stu_= 79 in *disc clin worse, and n*_Clin_=65 and *n*_Stu_= 79 in *worsening.* Blue bars/boxes indicate pre-POCT responses, green post-POCT responses. Significance denoted at: *p<0.05, **p<0.01, ***p<0.001.

As the WTS variables were not normally distributed (WTS-1: Shapiro-Wilk statistic (W)=0.860, df=560, p<0.001; WTS-2: Shapiro-Wilk statistic (W)=0.840, df=560, p<0.001), intragroup differences (pre-POCT vs. post-POCT) were analysed using Wilcoxon-Signed Rank tests and inter-group differences (clinicians vs. students) using Mann-Whitney U-tests.

**G. Mixed Effects Ordinal Logistic Regression- The effect of patient trajectory, initial WTS and in/voluntary POCT on final WTS**

Mixed effects ordinal logistic regression confirmed the findings of the linear regression: students were significantly less inclined to stop (despite receiving a negative POCT) when the patient’s trajectory was ambiguous (discordant scenarios) or worsening, as opposed to improving (odds ratio (OR) [95% confidence interval] = 0.62 [0.43-0.90], p=0.011). Final WTS was also a function of the student’s initial leaning, with high [low] WTS pre-POCT predicting high [low] WTS post-POCT (OR 1.51 [1.39-1.65], p<0.001). Finally, the manner in which the POCT result was acquired (voluntarily vs. involuntarily) did not influence students’ final WTS (OR 1.36 [0.82-2.25], p=0.25).

**H. The Effect of a Positive POCT on Confident Stop Decisions**

After learning that the POCT result had changed from negative to positive (*improvement* only), the WTS decreased significantly (clinicians: median [IQR]: 5.0[2.0] vs. 3.0[9.0]; z=-6.1, p<0.001; students: 4.0[2.0] vs. -2.0[6.0]; z=-6.5, p<0.001).

**I. Reasons for Stopping Antibiotics Per Scenario**

**
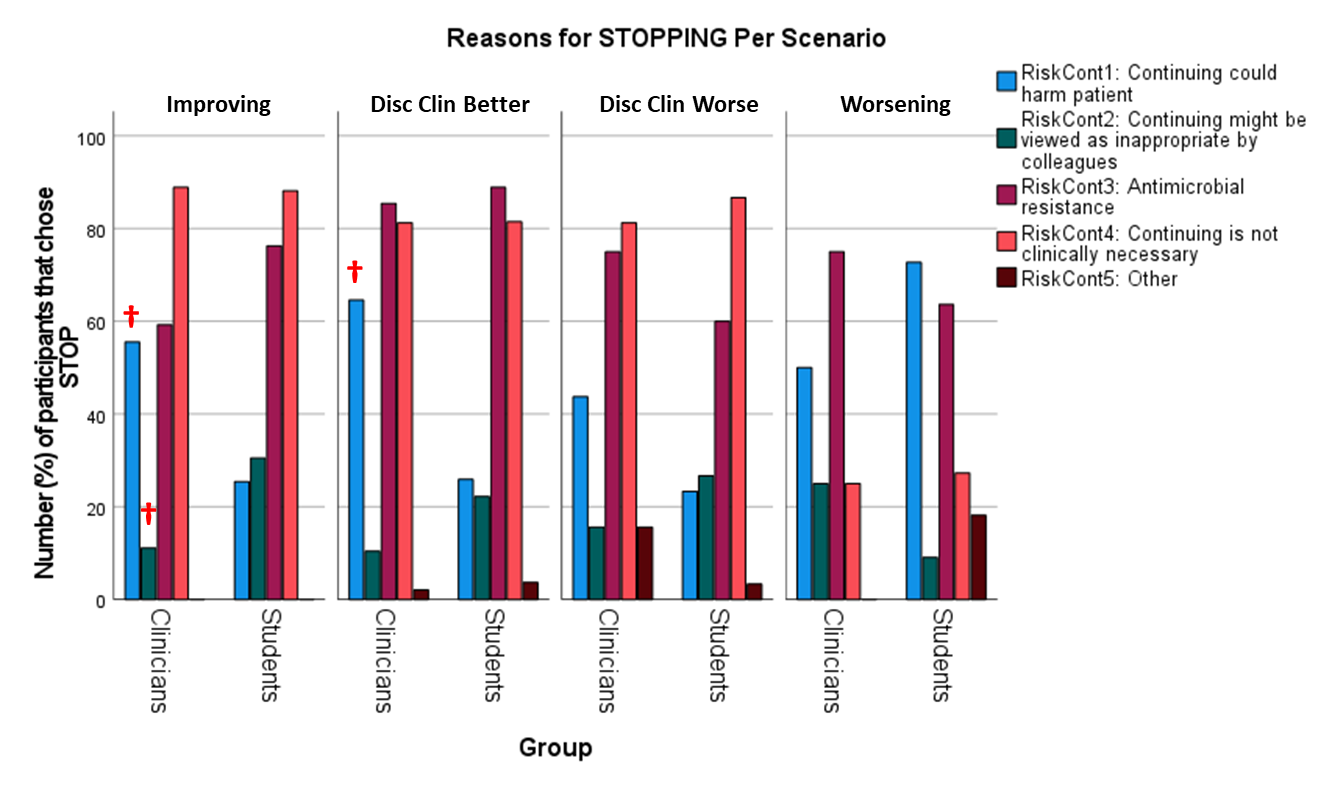
**

***Appendix I. Comparison of the percentage clinician and student reasons for stopping antibiotics, per scenario.*** The total number of clinician and student responses was *N*_Clin_=258 and *N*_Stu_=302; *n*_Clin_=62 and *n*_Stu_= 69 in *improving*, *n*_Clin_=66 and *n*_Stu_= 75 in *disc clin better*, *n*_Clin_=65 and *n*_Stu_= 79 in *disc clin worse, and n*_Clin_=65 and *n*_Stu_= 79 in *worsening. Participants could select multiple reasons for their decision. Differences in proportions (clinicians vs. students) were analysed using cluster-adjusted chi-square analysis. † Denotes significance between clinician and student reasoning in a given scenario at p<0.01.*

Overall, the most common reasons for stopping in both groups were “continuing is not clinically necessary” (clinicians vs. students: 83% [114/138] vs. 81% [103/127], chi-square(1)=0.101, p=0.750 and concerns over antimicrobial resistance (clinicians vs. students: 72% [100/138] vs. 74% [94/127], chi-square(1)=0.0810, p=0.776). This was particularly true for “improving”, “disc clin better” and “disc clin worse” scenarios. However, clinicians were more concerned than students about posing harm to the patient (56% [77/138] vs. 29% [37/127]; chi-square(1)=19.2, p<0.001), while students were more concerned than clinicians about their actions being viewed inappropriate by colleagues (26% [33/127] vs. 12% [17/138]; chi-square(1)=8.07, p=0.005). Looking at reasons for antibiotic decisions within each scenario we see that: clinicians (vs. students) were more concerned about posing harm to the patient in the two clinically improving scenarios, i.e. “improving” (56% [30/54] vs. 25% [15/59]; chi-square(1)=10.7, p=0.001) and “disc clin better” (65% [31/48] vs. 26% [7/27]; chi-square(1)=10.3, p=0.001), but not in the two clinically worsening scenarios (both p³0.090. Students (vs. clinicians) were more concerned about their actions being viewed inappropriate by colleagues in *improving* only (31% [18/59] vs. 25% [16/54]; chi-square(1)=6.34, p=0.012).

**J. Reasons for Continuing Antibiotics Per Scenario.**

**
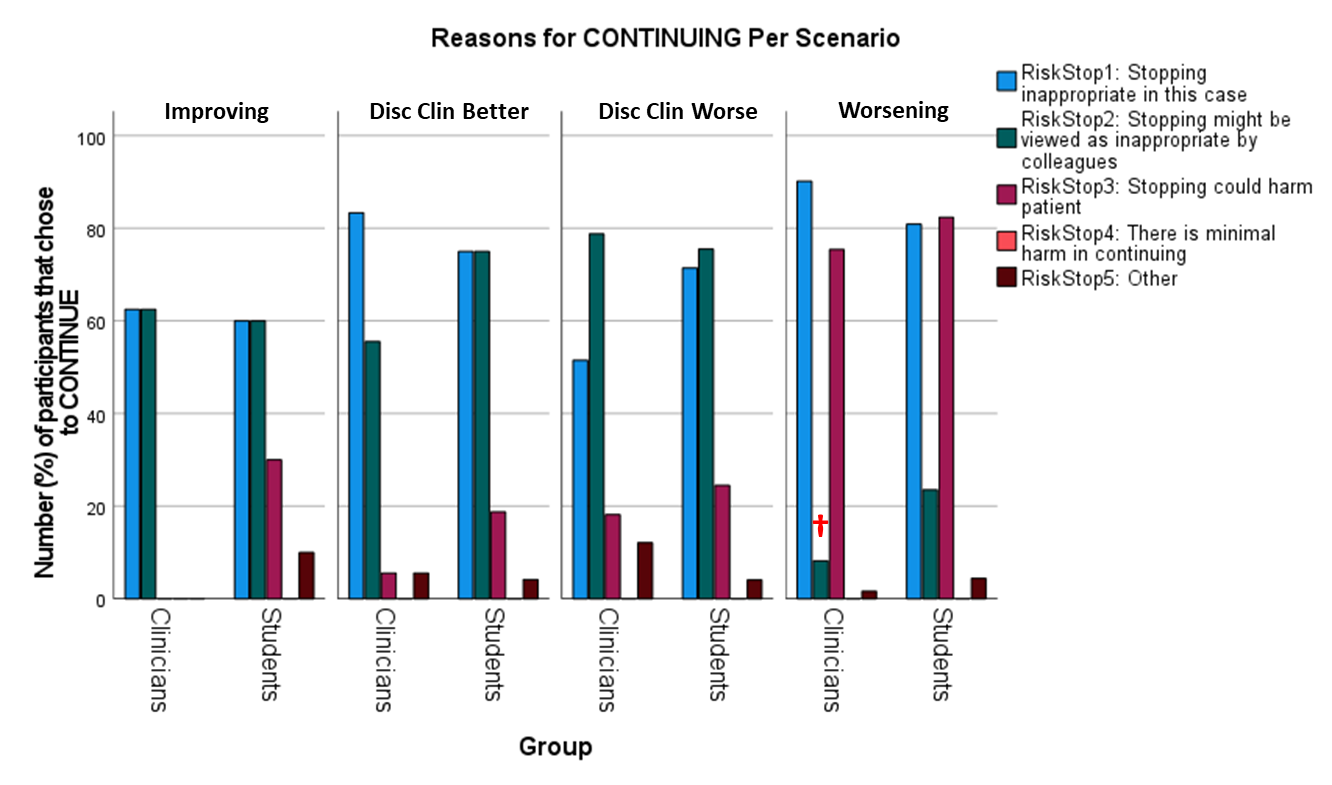
**

***Appendix J. Comparison of the percentage clinician and student reasons for continuing antibiotics, per scenario.*** The total number of clinician and student responses was *N*_Clin_=258 and *N*_Stu_=302; *n*_Clin_=62 and *n*_Stu_= 69 in *improving*, *n*_Clin_=66 and *n*_Stu_= 75 in *disc clin better*, *n*_Clin_=65 and *n*_Stu_= 79 in *disc clin worse, and n*_Clin_=65 and *n*_Stu_= 79 in *worsening. Participants could select multiple reasons for their decisions. Difference in proportions (clinicians vs. students) were analysed using cluster-adjusted chi-square analysis. † Denotes significance between clinician and student reasoning in a given scenario at p<0.01.*

Overall, the most common reason to continue antibiotics in both groups was “stopping is inappropriate in this case” (clinicians vs. students: 77% [92/120] vs. 75% [132/175], chi-square(1)=0.0600, p=0.807. However, once again, students were more concerned than clinicians about peers viewing their actions inappropriately (54% [95/175] vs. 38% [46/120]; chi-square(1)=10.7, p=0.007), which appeared to be true specifically in the “worsening” case, when scenarios were analysed separately.

**K. Reasons for Rejecting POCT Per Scenario.**

**
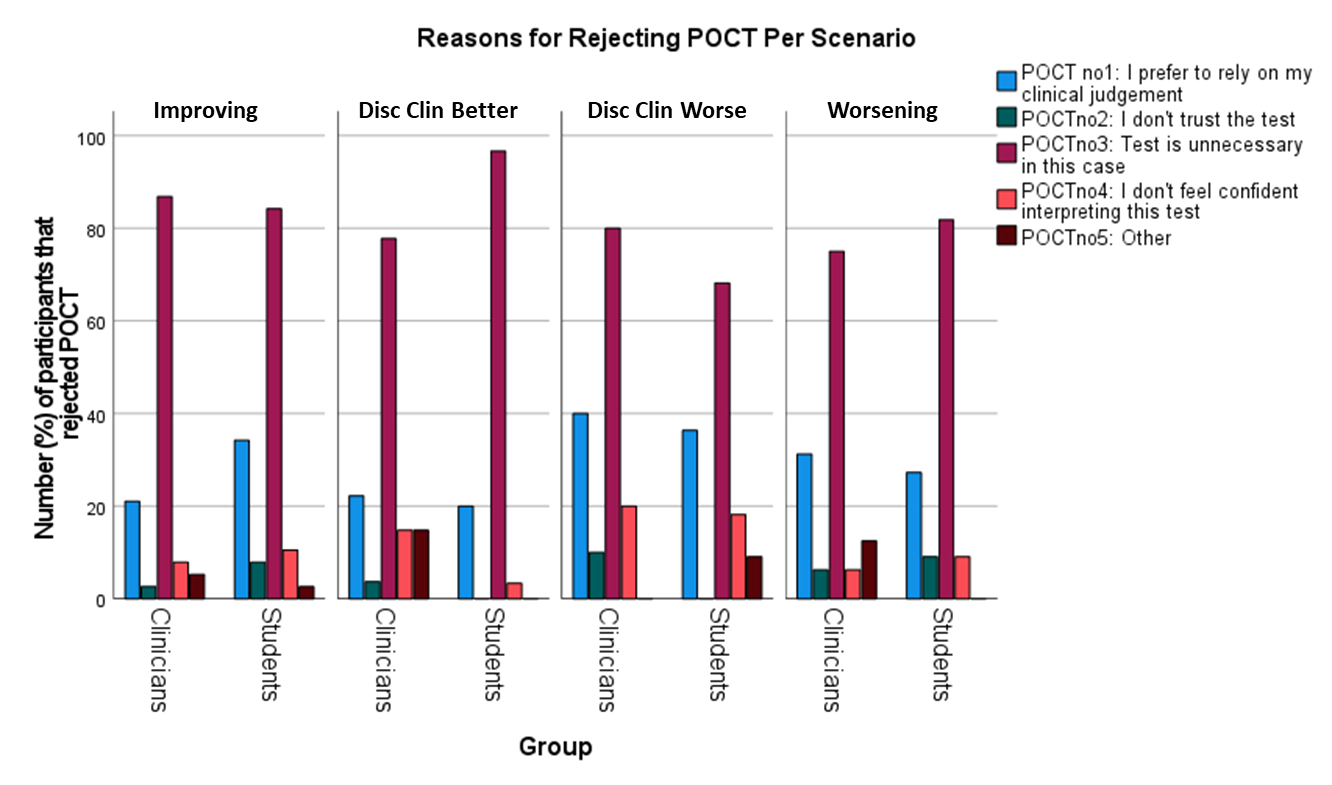
**

***Appendix K. Comparison of the percentage clinician and student reasons for rejecting POCT, per scenario****.* The total number of clinician and student responses was *N*_Clin_=258 and *N*_Stu_=302; *n*_Clin_=62 and *n*_Stu_= 69 in *improving*, *n*_Clin_=66 and *n*_Stu_= 75 in *disc clin better*, *n*_Clin_=65 and *n*_Stu_= 79 in *disc clin worse, and n*_Clin_=65 and *n*_Stu_= 79 in *worsening. Participants could select multiple reasons for their decisions. Differences in proportions (clinicians vs. students) were analysed using cluster-adjusted chi-square analysis. There were no significant differences.*

**L. Reasons for Requesting POCT Per Scenario**


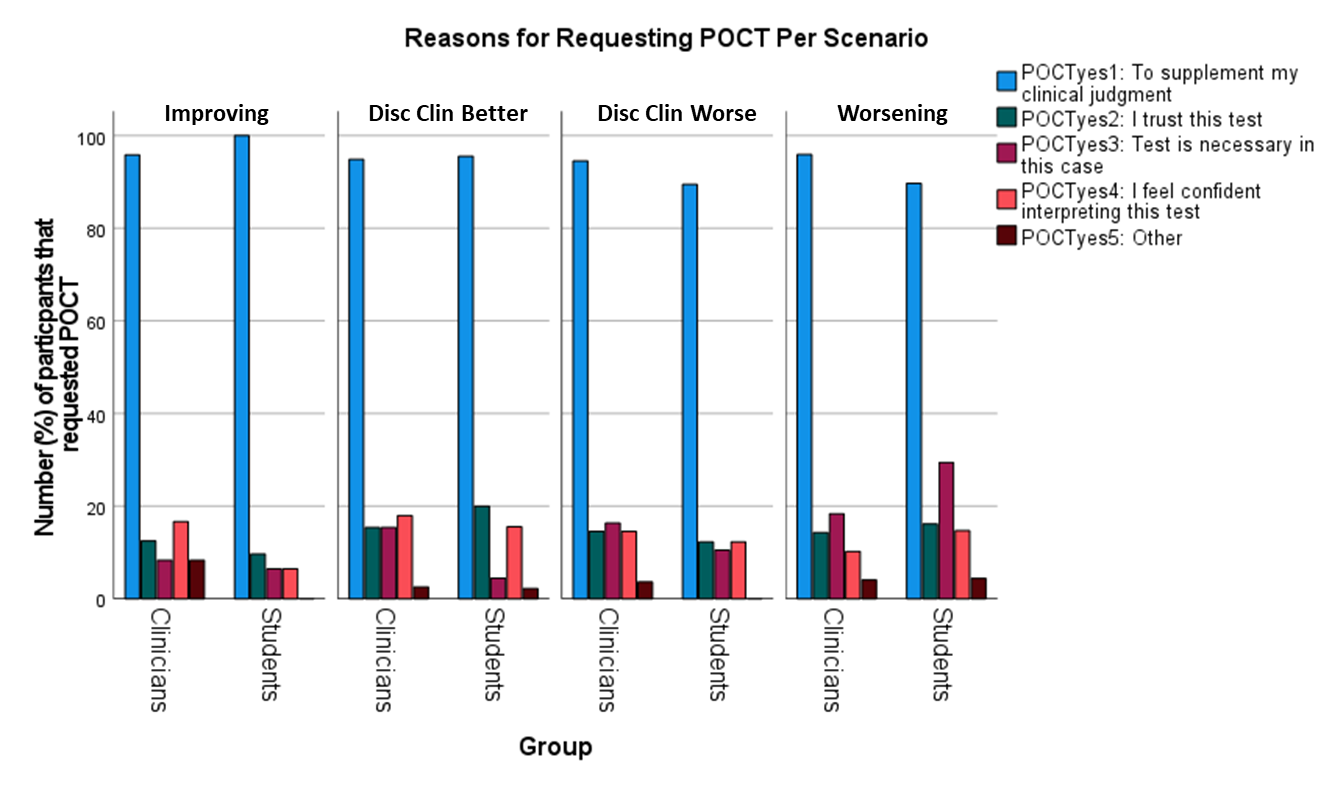
***Appendix L. Comparison of the percentage clinician and student reasons for requesting POCT, per scenario.*** The total number of clinician and student responses was *N*_Clin_=258 and *N*_Stu_=302; *n*_Clin_=62 and *n*_Stu_= 69 in *improving*, *n*_Clin_=66 and *n*_Stu_= 75 in *disc clin better*, *n*_Clin_=65 and *n*_Stu_= 79 in *disc clin worse, and n*_Clin_=65 and *n*_Stu_= 79 in *worsening. Participants could select multiple reasons for their decisinos. Difference in proportions (clinicians vs students) analysed using cluster-adjusted chi-square analysis. There were no significant differences.*

**M. Information given to Medical Students prior to completing the survey**


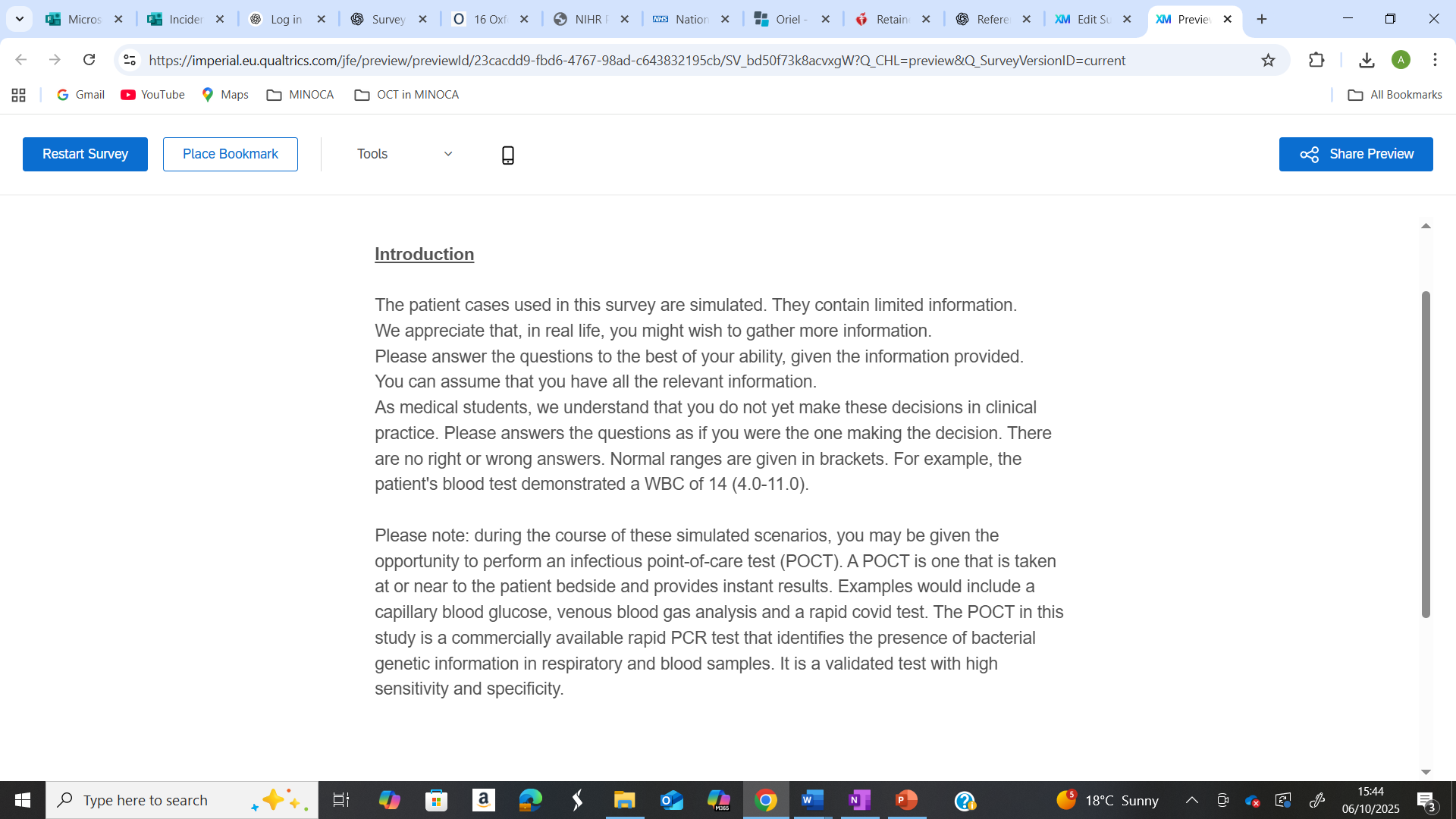

Supplement: dkaf486_Supplementary_Data [file dkaf486_supplementary_data.docx]
